# Insight into skin cell-based osteogenesis: a review

**DOI:** 10.12688/f1000research.10280.1

**Published:** 2017-03-17

**Authors:** Tingliang Wang, Lian Zhu, Ming Pei

**Affiliations:** 1 Stem Cell and Tissue Engineering Laboratory, Department of Orthopaedics, West Virginia University, Morgantown, WV, USA; 2 Department of Plastic and Reconstructive Surgery, Shanghai Ninth People’s Hospital Affiliated to Shanghai Jiao Tong University School of Medicine, Shanghai, China; 3 Division of Exercise Physiology, West Virginia University, Morgantown, WV, USA; 4 Mary Babb Randolph Cancer Center, Robert C. Byrd Health Sciences Center, West Virginia University, Morgantown, WV, USA

**Keywords:** stem cell therapy, skin cells, osteogenesis

## Abstract

For decades, researchers have been fascinated by the strategy of using cell therapy for bone defects; some progress in the field has been made. Owing to its ample supply and easy access, skin, the largest organ in the body, has gained attention as a potential source of stem cells. Despite extensive applications in skin and nerve regeneration, an increasing number of reports indicate its potential use in bone tissue engineering and regeneration. Unfortunately, few review articles are available to outline current research efforts in skin-based osteogenesis. This review first summarizes the latest findings on stem cells or progenitors in skin and their niches and then discusses the strategies of skin cell-based osteogenesis. We hope this article elucidates this topic and generates new ideas for future studies.

## Introduction

Finding appropriate therapeutic cells for bone regeneration has been a challenge for decades. Recently, stem cells from the skin, a potentially large cell source with easy access, have caught the attention of clinicians and scientists. More and more evidence indicates that skin stem cells are a potential cell source for bone regeneration. For example, heterozygous inactivating mutations of *GNAS* (encoding guanine nucleotide-binding G protein alpha subunit) cause diseases, including progressive osseous heteroplasia, Albright hereditary osteodystrophy, pseudohypoparathyroidism, and osteoma cutis ^1– 4^. These disorders have the common features of superficial ossification, starting with cutaneous ossification, with some involving subcutaneous and deeper tissues and some restricted to the skin. Multipotent progenitor cells and bone morphogenetic proteins (BMPs) were reported to be responsible for ectopic ossification ^5, 6^.

**Figure 1.**
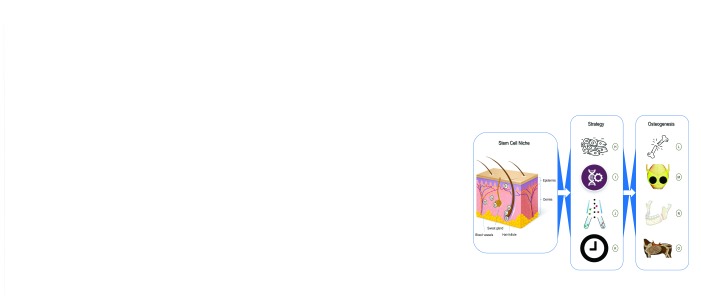
Skin cells for osteogenesis. (A–G) Stem cells and niches found in skin. (A) Hair follicle bulge-derived stem cells ^11, 12, 15^. (B) Hair follicle papilla-derived stem cells ^18, 22– 24^. (C) Hair sheath-derived stem cells ^16, 22^. (D) Pericytes ^10, 51^. (E) Sweat gland-derived stem cells ^25, 26^. (F) Interfollicle epidermis-derived stem cells ^13, 14^. (G) Stem cells from dermal niches that are not fully characterized ^27– 34, 50, 52, 53^. (H–K) Strategies for using skin cells. (H) Total skin fibroblasts ^35, 36^. (I) Genetic modification ^38– 48^. (J) Cell sorting ^33, 50– 53^. (K) Cell reprogramming ^56– 58, 65^. (L–O) Skin cells’ osteogenesis. (L) Limb bone defect regeneration ^35, 41, 42^. (M) Cranial bone defect regeneration ^38, 43, 44, 53^. (N) Mandibular bone defect regeneration ^40, 48^. (O) Rib bone defect regeneration ^45^.

Despite a decade of investigations using skin stem cells for regenerative medicine, most literature concerns their application in skin tissue engineering ^7^ and nerve regeneration ^8^, which was well covered by a recent review article ^9^. However, few review articles are available on skin cell-based osteogenesis. This review first summarizes the latest findings on stem cells or progenitors in skin and their niches and then discusses the strategies of skin cell-based osteogenesis ( Figure 1). We hope this article elucidates this topic and generates new ideas for future studies.

## Characteristics of skin stem cells and niches

Besides the primary structure of the epidermis, dermis, and subcutaneous tissue, there are hair follicles, vessels, capillaries, neurons, sweat glands, sebaceous glands, lymphatic capillaries, and erector pili muscles in skin, implying that there could be numerous niches for stem cells and progenitors in this tissue ( Table 1). Evidence also indicates that stem cells in skin, so-called pericytes, might be of perivascular origin ^10^.

**Table 1.** Characterization of skin stem cells and niches.

Location	Niche	Culture	Name	Markers	Differentiation potential	References
Epidermis	Interfollicle epidermis	Adherence	Epidermal stem cells	α6 integrin, β1 integrins, CD133, CD90, and keratin 15	Keratinocytes	13, 14
Hair follicle and appendages	Hair follicle bulge	Adherence	Keratinocyte stem cells/ epidermal neural crest stem cells	Keratin 15, keratin 19, β1 integrins, CD200, PHLDA1, follistatin, frizzled homolog 1, CD24 ^lo^, CD34 ^lo^, CD71 ^lo^, and CD146 ^lo^	Keratinocytes, all major neural crest lineages, including neurons, Schwann cells, myofibroblasts, melanocytes, and bone/ cartilage cells	11, 12, 14, 15
	Hair follicle sheath	Floating spheres	Dermal sheath cells	Nestin, fibronectin, CD34, and keratin 15(−)	Adipogenic and osteogenic lineages	16, 22
	Hair follicle papillae	Floating spheres	Skin-derived precursor cells	βIII-tubulin, p75NTR, NF-M; CNPase, GFAP, and S100β	Adipogenic, osteogenic, chondrogenic, and myogenic lineages, neurons, glia, and Schwann cells	18– 23
	Sweat gland	Adherence	Sweat gland stroma- derived stem cells	α6 integrin and nestin	Adipogenic, chondrogenic, and osteogenic lineages	25, 26
Dermis	Perivascular	Adherence	Pericytes	CD146, NG2, CD31(−), CD34(−), CD144(−), and VWF(−)	Adipogenic, chondrogenic, myogenic, and osteogenic lineages	10, 51
	Undefined niches of dermis	Adherence	Dermal stem cells/dermis- derived stromal cells	CD13, CD29, CD44, CD49d, CD71, CD73, CD90, CD105, CD166, SSEA4, vimentin, CD14(−), CD31(−), CD34(−), CD45(−), CD106(−), CD133(−), SSEA3(−), and nestin(−)	Adipogenic, chondrogenic, myogenic, and osteogenic lineages	27– 34, 50, 52, 53

CNPase, 2′,3′-cyclic nucleotide 3′-phosphodiesterase; GFAP, glial fibrillary acidic protein; NG2, neural/glial antigen 2; PHLDA1, pleckstrin homology-like domain family A member 1; SSEA4, stage-specific embryonic antigen-4; VWF, von Willebrand factor.

### Epidermis

Epidermal stem cells are found in both hair follicle bulge ^11, 12^ and interfollicular epidermis ^13, 14^. They are also viewed as keratinocyte stem cells because they generate cells that produce keratin ^11, 14^. Recent reports indicate that human epidermal stem cells are able to create all major neural crest derivatives containing neurons, Schwann cells, myofibroblasts, melanocytes, and bone/cartilage cells ^15, 16^. Despite the investigation of many stem cell markers, such as α6 integrin 5-bromo-2-deoxyuridine, β1 integrins, CD133, CD200, CD90, keratin 15, delta 1, and p63 ^17^, the molecular signature of epidermal stem cells remains undetermined.

### Hair follicle and appendages

Hair follicles have long been considered an important niche for stem cells because of the versatility in regeneration of hair and epidermis and wound repair. For example, skin-derived precursors (SKPs) from both murine and human origins residing in the papillae of hair follicles ^18^ can differentiate into neuron, glia, smooth muscle, and adipose cells ^19, 20^. As non-adherent cells, the SKPs are cultured as floating spheres with a neural crest origin ^21^. Although lineage differentiation crosses both ectoderm and mesoderm ^18, 20^, their potential for osteogenesis has seldom been tested, although a cell subpopulation characterized from hair follicle dermal papilla and dermal sheath of both rats and humans has the capacity for adipogenesis, myogenesis, chondrogenesis, and osteogenesis ^22– 24^. In addition, since keratinocytes can be generated from the hair follicle bulge, the hair follicle is an important niche for epidermal stem cells ^11, 12^. These findings indicate that the hair follicle is one of the most important niches in skin with stem cells and progenitors generating mesenchymal lineages. Recent studies indicate that sweat glands, a skin appendage, are also characterized as a niche for stem cells which can be isolated and induced into three mesodermal lineages ^25, 26^.

### Dermis

Dermis constitutes the majority of skin in both thickness and cell number. Dermal fibroblasts, the principal cells in dermis, have long been considered terminally differentiated cells and served as a negative control of mesenchymal stem cells (MSCs). When preserved in saline at 4°C for 6 days before digesting, non-hair follicle human dermis has been successfully proven to be an MSC source, indicative of a potential niche for stem cells ^27^. This finding is supported by another report, in which clonal analysis of a single dermal fibroblast isolated from human foreskin exhibited tripotent, bipotent, and unipotent ability ^28^, indicating multiple differentiation potential in dermal fibroblasts. Increasing evidence also demonstrates that these cells are positive for surface markers CD29, CD44, CD73, CD90, CD105, and CD166, indicating their MSC nature, and negative for CD14, CD31, CD34, CD45, and CD133, indicating non-hematopoietic lineage ^29– 34^.

## Strategies for using skin cells for osteogenesis

Fibroblasts from rabbit skin were osteoinduced followed by seeding on porous titanium pylon; this construct exhibited enhanced osseointegrative properties compared with unseeded pylon in both *in vitro* and *in vivo* studies ^35^. This study and others ^36^ suggest the possibility of using skin fibroblasts for osteogenesis, although an early report showed the inhibition of rat skin fibroblasts on mineralization of bone marrow MSCs ^37^. Unfortunately, owing to the low osteogenic potential of total skin fibroblasts with mixed cell populations, this kind of trial is far from successful. Therefore, it is critical to isolate skin cells with a preference for differentiation toward osteogenesis.

### Genetic modification

Using modification of genes to increase the expression of specific osteogenesis-related genes, skin fibroblasts, acting as “protein secretors” without differentiating by themselves or having the paracrine/exosomal effects that are found in MSCs, were promoted for bone tissue engineering and regeneration ^38– 41^. These genes of interest include *BMP-2* ^41– 45^, *BMP-4* ^42^, *BMP-7* ^38, 42^, *Runx2* (runt-related transcription factor 2) ^39, 43, 46, 47^, and *LMP-3* ( *lim mineralization protein-3*) ^40, 48^. In *in vivo* studies using skin fibroblasts, both ectopic osteogenesis and orthotopic bone regeneration are achieved through gene therapy ^42, 44^ from small animals like mice ^44^, rats ^38, 42, 48^, and rabbits ^41^ to large animals like equines ^45^. A study comparing different genes of interest for modification efficiency of skin fibroblasts determined that *BMP-2* is more powerful than *Runx2* ^43^ and that the mineralization ability of *Runx2*-modified skin fibroblasts is scaffold-dependent ^39^. Gene therapy is a promising method with a prominent effect; however, the safety of viral genetic modification needs further characterization ^49^.

### Cell sorting

Mixed populations isolated from total skin make cell therapy strategies for osteogenesis unsuccessful. Consequently, there are increasing efforts in sorting cells from skin to get target subpopulations. For example, type IV collagen-coated dishes have been used to attract CD29(+) human dermal stem cells via adherence, which exhibited higher osteogenic, adipogenic, and chondrogenic capacity compared with unsorted cells ^33^. CD271(+) and CD146(+) cells isolated from human skin and CD73(−)CD105(+) cells isolated from mouse skin by immunosorting also showed elevated multi-differentiation potential ^50– 52^. Interestingly, subpopulations sorted by other markers from human skin, such as CD73, stage-specific embryonic antigen-4 (SSEA-4), and BmprIB, show relatively restricted differentiation potential. For instance, BmprIB(+) cells can generate only an osteogenic lineage ^50, 53^, indicating that these subpopulations can be applied as therapeutic cells for osteogenesis because of their established lineage preference. However, concern due to low harvest rate resulting from cell sorting still exists ^50, 51, 53^.

### Cell reprogramming

Characterized by unlimited proliferation and differentiation potential like embryonic stem cells ^54, 55^, induced pluripotent stem cells (iPSCs) can be used in numerous stem cell therapies. As skin fibroblasts are the most abundant and easily accessed cells, they are commonly chosen as the parent cells of iPSCs. It has been well characterized that iPSC-derived osteoblasts can form osteoid both *in vitro* and *in vivo* ^56– 58^. A recent study revealed that bone defect repair is also achieved by human iPSCs in a radial defect model of immune-deficient mice ^59^. Furthermore, the involvement and mechanism of microRNAs in the regulation of mouse iPSCs during osteogenic differentiation have been preliminarily investigated ^60^.

## Conclusions and perspectives

In past decades, investigations using skin cells for osteogenesis have achieved significant progress. Many niches for stem cells in skin have been revealed and preliminarily characterized. Also, skin cells, enriched or not enriched, modified or not modified, are used for osteogenesis *in vitro* and *in vivo* and have achieved success in limb, cranial, mandibular, and rib bone defect regeneration ( Figure 1). However, some key problems remain unsolved. For example, since the niche for stem cells in dermis is not completely characterized, the efficiency of enriching stem cells or progenitors from skin is still restricted. For cell modification strategies, like gene therapy and cell reprogramming, the efficacy might be readily apparent, but the safety needs more in-depth research.

Recent developments in epigenetic conversion may shed some light on cell reprogramming. Unlike in iPSCs, epigenetic conversion does not completely reverse cells to the pluripotent stem cell stage ^61– 64^. This approach may avoid undesired side effects such as teratoma, which often occurs in the application of iPSCs and embryonic stem cells. Epigenetic conversion has achieved progress in directing fibroblasts from human skin and mouse embryos into cardiomyocytes, neuronal cells, and insulin-secreting cells with a mature phenotype ^61, 63, 64^. Although not much is known about converting skin fibroblasts into osteoblasts, there is a report of converting non-osteogenic cells into osteoblasts by epigenetic stimulation of *BMP-2* expression ^65^. By transient use of platelet-derived growth factor-AB and 5-azacytidine, mature bone and fat cells can also be converted into multipotent stem cells ^62^. Thus, although there are no studies characterizing the cells converted for bone regeneration, the most common candidate for epigenetic conversion, skin cells, may play a significant role in this strategy.

Taken together, two of these strategies are promising. One strategy is the enrichment of stem cells and progenitors from different skin niches. By improving the current low-efficiency cell isolation, a mass of therapeutic cells can be gathered from skin for better bone tissue engineering and regeneration. The other strategy is based on the easy access and abundant amount of skin fibroblasts. Via modification of the cell, either through iPSCs or the recent concept of epigenetic conversion, a differentiation-specific cell population can be manipulated and gathered. In that case, therapeutic cells for osteogenesis can be harvested on a large scale, making both the autologous and allogeneic approaches possible.
